# Gene expression of sternohyoid and diaphragm muscles in type 2 diabetic rats

**DOI:** 10.1186/1472-6823-13-43

**Published:** 2013-10-07

**Authors:** Erik van Lunteren, Michelle Moyer

**Affiliations:** 1 Pulmonary, Critical Care & Sleep Division, Department of Medicine, Louis Stokes, Cleveland, USA; 2 Department of Veterans Affairs Medical Center, Cleveland, OH 44106, USA; 3 Case Western Reserve University, Cleveland, OH 44106, USA

## Abstract

**Background:**

Type 2 diabetes differs from type 1 diabetes in its pathogenesis. Type 1 diabetic diaphragm has altered gene expression which includes lipid and carbohydrate metabolism, ubiquitination and oxidoreductase activity. The objectives of the present study were to assess respiratory muscle gene expression changes in type 2 diabetes and to determine whether they are greater for the diaphragm than an upper airway muscle.

**Methods:**

Diaphragm and sternohyoid muscle from Zucker diabetic fatty (ZDF) rats were analyzed with Affymetrix gene expression arrays.

**Results:**

The two muscles had 97 and 102 genes, respectively, with at least ± 1.5-fold significantly changed expression with diabetes, and these were assigned to gene ontology groups based on over-representation analysis. Several significantly changed groups were common to both muscles, including lipid metabolism, carbohydrate metabolism, muscle contraction, ion transport and collagen, although the number of genes and the specific genes involved differed considerably for the two muscles. In both muscles there was a shift in metabolism gene expression from carbohydrate metabolism toward lipid metabolism, but the shift was greater and involved more genes in diabetic diaphragm than diabetic sternohyoid muscle. Groups present in only diaphragm were blood circulation and oxidoreductase activity. Groups present in only sternohyoid were immune & inflammation and response to stress & wounding, with complement genes being a prominent component.

**Conclusion:**

Type 2 diabetes-induced gene expression changes in respiratory muscles has both similarities and differences relative to previous data on type 1 diabetes gene expression. Furthermore, the diabetic alterations in gene expression differ between diaphragm and sternohyoid.

## Background

Diabetes mellitus is one of the most rapidly growing chronic diseases of our time, with human type 2 diabetes becoming more prevalent than type 1 diabetes due to factors such as physical inactivity and increased obesity. Associated with the increasing prevalence of obesity and type 2 diabetes is the growing problem of obstructive sleep apnea and its adverse cardiovascular and neuropsychiatric consequences. Upper airway respiratory muscles are critical for the maintenance of pharyngeal patency during wakefulness and sleep, and for the restoration of pharyngeal patency when obstructive apneas occur during sleep. Many studies in humans and animal models of diabetes have confirmed reduced strength and endurance in respiratory and other skeletal muscles, [1-3] which reduces exercise performance and increases dyspnea [4-6]. Interestingly, upper airway muscle contractile properties are affected less than those of the diaphragm by type 1 diabetes, [3,7] although comparable data in type 2 diabetes are lacking.

Several cellular mechanisms underlying limb muscle adverse contractile changes have been identified from biochemical and electrophysiological studies in animal models of diabetes [8-11]. With respect to respiratory muscles, in type 1 diabetic diaphragm the expression of metabolism genes shifted by a small decrease in lipid metabolism gene expression and a large increase in carbohydrate metabolism gene expression; in addition there was increased expression of protein ubiquitination genes (a mechanism of protein breakdown), and increased expression of oxidoreductase genes (indicative of oxidative stress) [12]. It is unclear if type 2 diabetes affects gene expression of the respiratory muscles in the same manner as type 1 diabetes. Furthermore, it is unknown whether upper airway muscles are affected by diabetes in a similar manner as the diaphragm. However it is known from gene expression studies that compared with the diaphragm, the sternohyoid muscle has higher expression of carbohydrate metabolism genes, as well as lower expression of lipid metabolism genes, especially those involved directly in fatty acid β oxidation and biosynthesis in the mitochondria [13]. The hypothesis of the present study is that type 2 diabetes produces substantial changes in gene expression of the upper airway muscles, which furthermore differs both qualitatively and quantitatively from those of the diaphragm.

## Methods

All studies were approved by the institutional animal care and use committee and conformed with NIH guidelines for animal care. Studies were performed on 11 male Zucker Diabetic Fatty (ZDF) rats, an animal model of obesity and type 2 diabetes, obtained from Charles River Laboratories (Wilmington, MA). All animals had free access to food and water. Obese *(fa/fa)* animals (n=5) were fed Purina diet #5008, which induces development of type 2 diabetes between 8 and 12 weeks of age (Corsetti et al. 2000). Lean (+/?) littermates (n=6) were fed normal rodent chow. At an age of eighteen weeks, all animals were well-anesthetized with a mixture of intraperitoneal ketamine, xylazine and acepromazine following an all-night fast. Blood obtained from the tail was analyzed for glucose using a glucometer (Lifescan, Milpitas, CA). The entire sternohyoid and costal diaphragm muscles were removed surgically, placed in RNAlater, and stored at -80°C. At the time of muscle removal, fasting blood glucose values were 58 ± 7 mg/dl (range 36–77) for the normal animals, and 183 ± 60 mg/dl (range 133–275) for the obese ZDF animals (P < 0.001 by unpaired t test). The obese animals had a final weight that was heavier than the lean animals (424 ± 28 vs 348 ± 5 grams, for obese and lean, respectively, P < 0.02). Animals were not treated with insulin or oral hypoglycemics because the purpose of the study was to determine the effects of diabetes on gene expression rather than the extent to which treatment of diabetes would attenuate the changes.

Gene expression array studies were performed in a manner similar to that described previously [14-16]. Total RNA was extracted using Trizol (GibcoBRL, Rockville, MD), and the RNA pellets were resuspended at 1 μg RNA/μl DEPC-treated water. This was followed by a cleanup protocol with a Qiagen (Valencia, CA) RNeasy Total RNA mini kit. Total RNA was prepared using Affymetrix (Santa Clara, CA) microarrays, according to the directions from the manufacturer. Briefly, 8 μg of RNA was used in a reverse transcription reaction (SuperScript II; Life Technologies, Rockville, MD) to generate first strand cDNA. After second strand synthesis, double strand cDNA was used in an *in vitro* transcription reaction to generate biotinylated cRNA, which was purified and fragmented. Next, 15 μg of biotin-labeled cRNA was used in a 300 μl hybridization cocktail which included spiked transcript controls. 200 μl of cocktail was loaded onto Affymetrix RAE 230A microarrays (Santa Clara, CA) and hybridized for 16 hr at 45°C with agitation. Standard post-hybridization washes and double-stain protocols used an Affymetrix GeneChip Fluidics Station 400. Arrays were scanned using a Hewlett Packard Gene Array scanner, and analyzed with Affymetrix GCOS software. The data have been deposited in NCBIs Gene Expression Omnibus (GEO, http://www.ncbi.nlm.nih.gov/geo/query/acc.cgi?token=jjgprsaqewuayxo&acc=GSE21791) and assigned Series accession number GSE21791.

Statistical analysis was done with Bayesian analysis of variance for microarrays (BAM), using BAMarray software (http://www.bamarray.com) [17]. BAM balances the number of false detections against false non-detections by means of a special type of inferential regularization (i.e. borrowing strength across the data). Genes were further selected as significant based on consistent and appropriate present and absent calls in all samples per Affymetrix software. Subsequently signals were averaged for muscle from the lean and obese animals, and fold changes were calculated based on average values from each group. Analysis focused on genes whose expression changed at least ±1.5 fold in obese compared with lean muscle. To assign biological meaning to the group of genes with changed expression, the subset of genes which met the above criteria was further analyzed with the Gene Ontology (GO) classification system, using DAVID software (http://david.abcc.ncifcrf.gov/) [18]. Over-representation of genes with altered expression within specific GO categories was determined using the one-tailed Fisher exact probability modified by the addition of a jackknifing procedure, which penalizes the significance of categories with very few (eg. one or two) genes and favors more robust categories with larger numbers of genes [19].

Real-time PCR (RT-PCR) was used to confirm changes in gene expression as described previously [14-16]. Testing was done using the same tissue that had been used for gene expression arrays, and was performed on genes which were chosen from the main, statistically over-represented, GO groupings based on biological interest. An Applied Biosystems ABI 7900HT unit with automation attachment (Foster City, CA) was used for real-time PCR. This unit is capable of collecting spectral data at multiple points during a PCR run. To execute the first step and make archive cDNA, 3 μg of total RNA were reverse transcribed in a 100 μl reaction using Applied Biosystems enzymes and reagents in accordance with the manufacturer’s protocols. RNA samples were accurately quantitated using a Nanodrop Technologies ND-1000 spectrophotometer (Wilmington, DE). Equal amounts of total RNA were reverse transcribed and then used in PCR amplifications. β-Actin had very little variation in expression across the sample set and therefore was chosen as the endogenous control. Since many of the target genes of interest were signaling molecules and likely to be expressed at low levels, we opted for a low dilution factor so as to create an environment more conducive to obtaining reliable results. The cDNA reaction from above was diluted by a factor of 10. For the PCR step, 9 μl of this diluted cDNA were used for each of three replicate 15 μl-reactions carried out in a 384 well plate. Standard PCR conditions were used for the Applied Biosystems assays: 50°C for 2 min, followed by 95°C for 10 min, followed by 40 cycles of 95°C for 15 sec alternating with 60°C for 1 min each. rtPCR analysis was similar to our previous studies^14-16^. Values for RNA abundance were normalized for each gene with respect to the endogenous control in that sample (β-Actin), mean values for fold changes were calculated for each gene, and statistical testing was performed with the unpaired t-test (two-tailed).

## Results

There were 54 genes with significantly increased expression and 43 genes with significantly reduced expression in diabetic compared with normal diaphragm, using the cut-off of at least a ± 1.5-fold changed expression in addition to consistent present calls by Affymetrix software and statistical significance by BAM. Using the same criteria, there were 50 genes with significantly increased expression and 52 genes with significantly reduced expression in diabetic compared to normal sternohyoid. A complete list of these genes, including mean fold change values for each gene, is provided in Additional file 1 (for online publication only). Classification of genes by Gene Ontology (GO) groups and statistical testing of over-representation among GO groups was done separately for each muscle for the genes with significantly changed expression.

Among the genes with at least 1.5-fold changed expression in diabetic diaphragm, assignment to GO groups was possible for 55 using the biological function classification, 61 using the molecular function classification, and 69 using the cellular constituent classification. In the diabetic sternohyoid, assignment to GO groups was possible for 66 using the biological function classification, 45 using the molecular function classification, and 58 using the cellular constituent classification. The GO terms with over-representation among these genes in the diaphragm and sternohyoid are indicated in Table 1.

**Table 1:** Statistically significant over-represented Gene Ontology (GO) terms to which genes with changed expression in diaphragm and sternohyoid of diabetic animals were assigned

***DIAPHRAGM***			
***GO classification***	***GO group***	***# genes***	***P value***
***Biological process***			
	Transport	27	0.000089
	Establishment of localization	27	0.00020
	Monocarboxylic acid metabolic process	8	0.00035
	Localization	29	0.00050
	Monosaccharide biosynthetic process	4	0.00067
	Alcohol biosynthetic process	4	0.00071
	Ion transport	12	0.0015
	Alcohol metabolic process	8	0.0018
	Hexose metabolic process	6	0.0036
	Monosaccharide metabolic process	6	0.0037
	Carboxylic acid metabolic process	9	0.0069
	Inorganic anion transport	5	0.0072
	Organic acid metabolic process	9	0.0073
	Regulation of ion transport	3	0.014
	Striated muscle contraction	3	0.014
	Anion transport	5	0.015
	Carbohydrate biosynthetic process	4	0.016
	Blood circulation	5	0.016
	Circulatory system process	5	0.017
	Regulation of biological process	28	0.018
	Lipid metabolic process	9	0.019
	Heart development	4	0.022
	Cardiac muscle development	2	0.027
	Cellular carbohydrate metabolic process	6	0.034
	Peptide transport	3	0.036
	Carbohydrate metabolic process	7	0.040
	Circadian rhythm	3	0.040
	Multicellular organismal development	18	0.042
	Regulation of multicellular organismal process	6	0.044
	Developmental process	23	0.045
	Regulation of transport	4	0.046
	Regulation of the force of heart contraction	2	0.047
	Long-chain fatty acid transport	2	0.048
***Cellular constituent***			
	Cytoplasm	42	0.0000080
	Cytoplasmic part	30	0.0014
	Fibrillar collagen	3	0.0015
	Sarcoplasmic reticulum	3	0.0047
	Intracellular part	50	0.0048
	Sarcoplasm	3	0.0065
	Sarcomere	4	0.0066
	Endosome	5	0.0066
	Plasma membrane	20	0.0072
	Endoplasmic reticulum	10	0.0097
	Myofibril	4	0.010
	Contractile fiber part	4	0.010
	Intracellular	52	0.011
	Collagen type I	2	0.013
	Contractile fiber	4	0.014
	Collagen	3	0.018
	Smooth endoplasmic reticulum	2	0.032
	Cell part	71	0.039
	Cell	71	0.039
***Molecular function***			
	Protein binding	39	0.0034
	Substrate specific channel activity	6	0.018
	Calcium ion binding	9	0.024
	Structural constituent of bone	2	0.026
	Passive transmembrane transporter activity	6	0.030
	Transporter activity	13	0.031
	Channel activity	6	0.031
	Auxiliary transport protein activity	3	0.032
	Substrate-specific transporter activity	11	0.033
	Oxidoreductase activity	10	0.035
	Binding	58	0.039
	Transmembrane transporter activity	10	0.044
***STERNOHYOID***			
***GO classification***	***GO group***	***# genes***	***P value***
***Biological process***			
	Regulation of multicellular organismal process	12	0.0000012
	Humoral immune response	6	0.0000075
	Complement activation	5	0.000014
	Activation of plasma proteins during acute inflammatory response	5	0.000016
	Acute inflammatory response	6	0.000027
	Regulation of immune response	6	0.000027
	Regulation of immune system process	6	0.000036
	Activation of immune response	5	0.000070
	Immune effector process	6	0.000076
	Response to stress	17	0.000096
	Monocarboxylic acid metabolic process	9	0.00010
	Positive regulation of immune response	5	0.00013
	Positive regulation of immune system process	5	0.00016
	Carboxylic acid metabolic process	12	0.00021
	Innate immune response	5	0.00025
	Organic acid metabolic process	12	0.00078
	Positive regulation of multicellular organismal process	5	0.00091
	Long-chain fatty acid transport	3	0.0012
	Muscle contraction	6	0.0015
	Muscle system process	6	0.0017
	Fatty acid transport	3	0.0021
	Regulation of muscle contraction	4	0.0022
	Response to hormone stimulus	6	0.0028
	Fatty acid metabolic process	6	0.0030
	B cell mediated immunity	4	0.0032
	Immune response	9	0.0033
	Anion transport	6	0.0037
	Lymphocyte mediated immunity	4	0.0048
	Adaptive immune response based on somatic recombination of immune receptors built from immunoglobulin superfamily domains	4	0.0057
	Adaptive immune response	4	0.0062
	Humoral immune response mediated by circulating immunoglobulin	3	0.0064
	Complement activation-classical pathway	3	0.0064
	Defense response	8	0.0067
	Leukocyte mediated immunity	4	0.0071
	Response to biotic stimulus	6	0.0074
	Lipid transport	4	0.0077
	Inflammatory response	6	0.0082
	Positive regulation of biological process	13	0.0082
	Response to external stimulus	10	0.0084
	Ion transport	11	0.0089
	Inorganic anion transport	5	0.010
	Cellular lipid metabolic process	9	0.010
	Response to unfolded protein	4	0.011
	Response to protein stimulus	4	0.011
	Lipid metabolic process	10	0.012
	Immune system process	10	0.013
	Metabolic process	53	0.015
	Response to steroid hormone stimulus	4	0.022
	Response to wounding	7	0.023
	Response to endogenous stimulus	7	0.028
	Response to corticosteroid stimulus	3	0.029
	Regulation of Wnt receptor signaling pathway	3	0.030
	Response to peptide hormone stimulus	3	0.037
	Regulation of biological process	28	0.038
	Cellular metabolic process	45	0.049
***Cellular constituent***			
	Cytoplasm	45	9.2E-07
	Extracellular region part	25	0.000068
	Proteinaceous extracellular matrix	9	0.000087
	Extracellular matrix	9	0.00010
	Extracellular region	25	0.00017
	Extracellular space	23	0.00030
	Collagen	4	0.0013
	Cytoplasmic part	31	0.00140
	Fibrillar collagen	3	0.0016
	Extracellular matrix part	5	0.00230
	Intracellular part	52	0.0040
	Endoplasmic reticulum	10	0.0080
	Intracellular	54	0.011
	Complement component C1 complex	2	0.012
	Collagen type I	2	0.012
	Glycerol-3-phosphate dehydrogenase complex	2	0.018
	Organelle outer membrane	3	0.033
	Envelope	7	0.034
	Organelle envelope	7	0.036
***Molecular function***			
	Glutathione transferase activity	4	0.00076
	Catalytic activity	43	0.00090
	Calcium ion binding	12	0.0033
	Transferase activity- transferring alkyl or aryl (other than methyl) groups	4	0.0042
	Glycerol-3-phosphate dehydrogenase activity	2	0.012
	Transferase activity	17	0.024
	Structural constituent of bone	2	0.029
	Magnesium ion binding	5	0.037

The identified GO groups varied considerably with respect to number of constituent genes (ranging from 2 to 71) and degree of specificity (e.g., from specific terms such as fatty acid transport and glycerol-3-phosphate dehydrogenase activity to general terms such as cell and binding). The more specific GO groups were chosen for further analysis; in many instances there were clusters of closely related GO groups that were considered together (Table 2). Themes common to both muscles were lipid metabolism, carbohydrate metabolism, muscle contraction, ion transport and collagen. Themes present in only diaphragm were blood circulation and oxidoreductase activity. Themes present in only sternohyoid were immune & inflammation and response to stress & wounding.

**Table 2:** Specific Gene Ontology groups which were examined in more detail to which genes with changed expression in diaphragm and sternohyoid of diabetic animals were assigned

***GO group category & specific GO term***	***Diaphragm # genes***	***P value***	***Sternohyoid # genes***	***P value***
***Metbolism - Lipid***				
Lipid metabolic process	9	0.019	10	0.012
Long-chain fatty acid transport	2	0.048	3	0.0012
Cellular lipid metabolic process			9	0.010
Fatty acid metabolic process			6	0.0030
Lipid transport			4	0.0077
Fatty acid transport			3	0.0021
***Metabolism - Carbohydrate***				
Carbohydrate metabolic process	7	0.040		
Monosaccharide metabolic process	6	0.0037		
Hexose metabolic process	6	0.0036		
Cellular carbohydrate metabolic process	6	0.034		
Carbohydrate biosynthetic process	4	0.016		
Glycerol-3-phosphate dehydrogenase complex			2	0.018
Glycerol-3-phosphate dehydrogenase activity			2	0.012
***Muscle contraction***				
Sarcomere	4	0.0066		
Myofibril	4	0.010		
Contractile fiber part	4	0.010		
Contractile fiber	4	0.014		
Striated muscle contraction	3	0.014		
Muscle system process			6	0.0017
Muscle contraction			6	0.0015
Regulation of muscle contraction			4	0.0022
***Ion transport***				
Ion transport	12	0.0015	11	0.0089
Calcium ion binding	9	0.024	12	0.0033
Inorganic anion transport	5	0.0072	5	0.010
Anion transport	5	0.015	6	0.0037
Regulation of ion transport	3	0.014		
Channel activity	6	0.031		
Magnesium ion binding			5	0.037
***Collagen***				
Fibrillar collagen	3	0.0015	3	0.0016
Collagen	3	0.018	4	0.0012
Collagen type I	2	0.013	2	0.012
***Blood circulation***				
Blood circulation	5	0.016		
Circulatory system process	5	0.017		
***Oxidoreductase activity***				
Oxidoreductase activity	10	0.035		
***Immune & inflammatory***				
Immune system process			10	0.013
Immune response			9	0.0033
Defense response			8	0.0067
Immune effector process			6	0.000076
Acute inflammatory response			6	0.000027
Regulation of immune system process			6	0.000036
Inflammatory response			6	0.0082
Humoral immune response			6	7.5E-06
Regulation of immune response			6	0.000027
Activation of immune response			5	0.000070
Positive regulation of immune system process			5	0.00016
Complement activation			5	0.000014
Positive regulation of immune response			5	0.00013
Innate immune response			5	0.00025
Adaptive immune response			4	0.0062
Leukocyte mediated immunity			4	0.0071
Lymphocyte mediated immunity			4	0.0048
Adaptive immune response			4	0.0057
B cell mediated immunity			4	0.0032
Humoral immune response immunoglobulin			3	0.0064
Complement activation-classical pathway			3	0.0064
Complement component C1 complex			2	0.012
***Response to stress & wounding***				
Response to stress			17	0.000096
Response to wounding			7	0.023

The genes in both the diaphragm and sternohyoid that were classified in either lipid or carbohydrate metabolism GO groups, as well as the direction and magnitude of their changed expression, are listed in Table 3. In the diaphragm there were 9 genes involved in lipid metabolism (6 increased/3 decreased) and 7 genes involved in carbohydrate metabolism (1 increased/6 decreased). In the sternohyoid, there were 10 genes involved in lipid metabolism (7 increased/3 decreased) and 2 genes involved in carbohydrate metabolism (0 increased/2 decreased). With respect to specific genes, there were only 3 genes that had changed expression in both diaphragm and sternohyoid in response to diabetes. Carnitine O-octanoyltransferase (Crot), which plays a role in fatty acid transport, was increased in both muscles. Glycerol-3-phosphate dehydrogenase 2 (Gpd2), a carbohydrate metabolism gene which is involved in gluconeogenesis, and Acyl-CoA synthetase (Acsl6), a major enzyme in fatty acid metabolism gene, were decreased in both tissues. For both muscles together, lipid metabolism gene expression was increased more than decreased (total 13 vs. 6 genes). On the other hand, for carbohydrate metabolism, there were more genes that had decreased expression than those that had increased expression (total 8 vs. 1 genes).

**Table 3:** Genes with changed expression in diabetic diaphragm and sternohyoid that were assigned to specific statistically over-represented Gene Ontology (GO) terms

***DIAPHRAGM***		
***Gene title***	***Gene symbol***	***FC***
***Lipid metabolism***		
Protein kinase, AMP-activated, alpha 1 catalytic subunit	Prkaa1	2.2
Cell death-inducing DNA fragmentation factor	Cidea	2.2
Diazepam binding inhibitor	Dbi	1.7
Carnitine O-octanoyltransferase	Crot	1.6
Adipose differentiation related protein	Adfp	1.6
Low density lipoprotein-related protein 1 (alpha-2-macroglobulin receptor)	Lrp1	1.5
Acyl-CoA synthetase long-chain family member 6	Acsl6	−1.7
Thyroid hormone responsive	Thrsp	−2.1
Transmembrane 7 superfamily member 2	Tm7sf2	−2.5
***Carbohydrate metabolism***		
Protein kinase, AMP-activated, alpha 1 catalytic subunit	Prkaa1	2.2
UDP-glucose pyrophosphorylase 2	Ugp2	−1.5
Solute carrier family 2 (facilitated glucose transporter), member 4	Slc2a4	−1.5
Coenzyme Q7 homolog, ubiquinone (yeast)	Coq7	−1.6
6-phosphofructo-2-kinase/fructose-2,6-biphosphatase 1	Pfkfb1	−1.7
Glycerol-3-phosphate dehydrogenase 2, mitochondrial	Gpd2	−1.7
Dicarbonyl L-xylulose reductase	Dcxr	−2.0
***Muscle contraction***		
Cysteine and glycine-rich protein 3	Csrp3	4.2
Myosin binding protein H	Mybph	3.0
PDZ and LIM domain 3	Pdlim3	1.6
Calsequestrin 2 (cardiac muscle)	Casq2	1.5
Myosin, heavy chain 4, skeletal muscle	Myh4	−3.6
***Ion transport***		
***Calcium channel***		
Myosin, light chain 6B, alkali, smooth muscle and non-muscle	Myl6b	6.8
Calcium channel, voltage-dependent, beta 2 subunit	Cacnb2	1.6
Sarcolipin	Sln	1.6
Calsequestrin 2 (cardiac muscle)	Casq2	1.5
Integrin alpha 7	Itga7	1.5
Glycerol-3-phosphate dehydrogenase 2, mitochondrial	Gpd2	−1.7
S100 calcium binding protein A3	S100a3	−1.7
Parvalbumin	Pvalb	−2.0
Phospholamban	Pln	−2.4
***Sodium/Potassium channels***		
Sodium channel, voltage-gated, type III, beta	Scn3b	2.2
Potassium large conductance calcium-activated channel subfamily M, alpha member 1	Kcnma1	1.5
FXYD domain-containing ion transport regulator 7	Fxyd7	−1.7
***Other channels***		
Low density lipoprotein-related protein 1 (alpha-2-macroglobulin receptor)	Lrp1	1.5
Solute carrier family 30 (zinc transporter), member 4	Slc30a4	1.5
Aquaporin 1	Aqp1	−1.5
Chloride channel 4-2	Clcn4-2	−1.8
***Collagen***		
Collagen, type I, alpha 2	Col1a2	−1.6
Collagen, type III, alpha 1	Col3a1	−1.7
Collagen, type I, alpha 1	Col1a1	−2.0
Ficolin (collagen/fibrinogen domain containing) 1	Fcn1	−2.1
***Collagen***		
Collagen, type I, alpha 2	Col1a2	−1.6
Collagen, type III, alpha 1	Col3a1	−1.7
Collagen, type I, alpha 1	Col1a1	−2.0
***Blood circulation***		
Cysteine and glycine-rich protein 3	Csrp3	4.2
Potassium large conductance calcium-activated channel subfamily M, alpha member 1	Kcnma1	1.5
Collagen, type III, alpha 1	Col3a1	−1.7
Apelin	Apln	−2.2
Phospholamban	Pln	−2.4
***Oxidoreductase activity***		
2,4-dienoyl CoA reductase 1, mitochondrial	Decr1	2.0
IMP (inosine monophosphate) dehydrogenase 2	Impdh2	1.6
Potassium large conductance calcium-activated channel subfamily M, alpha member 1	Kcnma1	1.5
Solute carrier family 30 (zinc transporter), member 4	Slc30a4	1.5
Coenzyme Q7 homolog, ubiquinone (yeast)	Coq7	−1.6
Glycerol-3-phosphate dehydrogenase 2, mitochondrial	Gpd2	−1.7
S100 calcium binding protein A3	S100a3	−1.7
Dicarbonyl L-xylulose reductase	Dcxr	−2.0
Phytanoyl-CoA dioxygenase domain containing 1	Phyhd1	−2.1
Transmembrane 7 superfamily member 2	Tm7sf2	−2.5
***STERNOHYOID***		
***Gene title***	***Gene Symbol***	***FC***
***Lipid metabolism***		
Acyl-CoA thioesterase 2	Acot2	2.4
Nudix (nucleoside diphosphate linked moiety X)-type motif 4	Nudt4	2.1
Retinol saturase (all trans retinol 13,14 reductase)	Retsat	1.9
Carnitine palmitoyltransferase 1b, muscle	Cpt1b	1.7
Carnitine O-octanoyltransferase	Crot	1.6
Carnitine palmitoyltransferase 2	Cpt2	1.5
Solute carrier family 27 (fatty acid transporter), member 1	Slc27a1	1.5
Acyl-CoA synthetase long-chain family member 6	Acsl6	−1.6
Thyroid hormone responsive	Thrsp	−2.1
Sterol regulatory element binding transcription factor 1	Srebf1	−2.4
***Carbohydrate metabolism***		
Glycerol-3-phosphate dehydrogenase 1 (soluble)	Gpd1	−1.6
Glycerol-3-phosphate dehydrogenase 2, mitochondrial	Gpd2	−1.8
***Muscle contraction***		
Myosin binding protein H	Mybph	8.6
Complement component 4a	C4a	2.0
Calsequestrin 2 (cardiac muscle)	Casq2	2.0
Cholinergic receptor, nicotinic, delta	Chrnd	1.9
Heat shock protein, alpha-crystallin-related, B6	Hspb6	−1.6
Guanidinoacetate N-methyltransferase	Gamt	−1.6
***Ion transport***		
***Calcium channel***		
S100 calcium-binding protein A4	S100a4	1.9
Complement component 1, q subcomponent, beta polypeptide	C1qb	1.7
Complement component 1, q subcomponent, alpha polypeptide	C1qa	1.6
Macrophage galactose N-acetyl-galactosamine specific lectin 1	Mgl1	1.5
Calsequestrin 2 (cardiac muscle)	Casq2	1.5
Follistatin-like 1	Fstl1	−1.5
Glycerol-3-phosphate dehydrogenase 2, mitochondrial	Gpd2	−1.7
Eukaryotic elongation factor-2 kinase	Eef2k	−2.0
ATPase, Ca++ transporting, plasma membrane 3, (AKA PMCA2)	Atp2b3	−2.1
Myosin, light chain 6B, alkali, smooth muscle and non-muscle	Myl6b	−2.5
***Sodium/Potassium channels***		
FXYD domain-containing ion transport regulator 2	Fxyd2	1.6
Potassium large conductance calcium-activated channel subfamily M, alpha member 1	Kcnma1	1.5
ATPase, Na+/K+ transporting, beta 2 polypeptide	Atp1b2	−1.6
***Magnesium***		
Inositol (myo)-1(or 4)-monophosphatase 2	Impa2	1.8
Acyl-CoA synthetase long-chain family member 6	Acsl6	−1.6
***Other channels***		
Latent transforming growth factor beta binding protein 1	Ltbp1	2.0
Cholinergic receptor, nicotinic, delta	Chrnd	1.9
2,4-dienoyl CoA reductase 2, peroxisomal	Decr2 /// Rab11fip3	−1.6
Solute carrier family 16, member 3 (monocarboxylic acid transporter 4)	Slc16a3	−1.7
Amylase, alpha 1A (salivary)	Amy1a	−1.7
***Collagen***		
Collagen, type V, alpha 1	Col5a1	−1.8
Collagen, type I, alpha 1	Col1a1	−1.9
Collagen, type I, alpha 2	Col1a2	−2.2
***Collagen***		
Collagen, type V, alpha 1	Col5a1	−1.8
Collagen, type XV, alpha 1	Col15a1	−1.9
Collagen, type I, alpha 1	Col1a1	−1.9
Collagen, type I, alpha 2	Col1a2	−2.2
***Immune & inflammatory***		
Cyclin-dependent kinase inhibitor 1A (p21, Cip1)	Cdkn1a	2.4
Fc fragment of IgG, low affinity IIb, receptor (CD32)	Fcgr2b	2.2
Complement component 4a	C4a	2.0
Adipsin	Adn	1.8
Complement component 1, q subcomponent, beta polypeptide	C1qb	1.7
Complement component 1, q subcomponent, alpha polypeptide	C1qa	1.6
Complement factor H	Cfh	1.6
Dipeptidylpeptidase 4	Dpp4	−1.9
Myxovirus (influenza virus) resistance 1	Mx1	−1.9
Spondin 2, extracellular matrix protein	Spon2	−2.0
***Response to stress & wounding***		
Cyclin-dependent kinase inhibitor 1A (p21, Cip1)	Cdkn1a	2.4
Acyl-CoA thioesterase 2	Acot2	2.4
Fc fragment of IgG, low affinity IIb, receptor (CD32)	Fcgr2b	2.2
Complement component 4a	C4a	2.0
Deleted in malignant brain tumors 1	Dmbt1	1.9
Complement factor D (adipsin)	Adn	1.8
Complement component 1, q subcomponent, beta polypeptide	C1qb	1.7
Potassium large conductance calcium-activated channel subfamily M, alpha member 1	Kcnma1	1.6
Complement component 1, q subcomponent, alpha polypeptide	C1qa	1.6
Complement factor H	Cfh	1.6
Epidermal growth factor receptor	Egfr	−1.5
SRY (sex determining region Y)-box 4	Sox4	−1.6
Serine (or cysteine) peptidase inhibitor, clade H, member 1	Serpinh1	−1.6
Heat shock protein, alpha-crystallin-related, B6	Hspb6	−1.6
Collagen, type I, alpha 1	Col1a1	−1.9
Heat shock protein 2	Hspa2	−2.0
Sterol regulatory element binding transcription factor 1	Srebf1	−2.4

Fold changes (FC) in gene expression are represented in the last column.

There were 5 muscle contraction genes with significantly changed expression in the diaphragm (4 increased/1 decreased) and 6 with significantly changed expression in the sternohyoid (4 increased/2 decreased) with diabetes (Table 3). Of note is that myosin binding protein H (Mybph) and calsequestrin 2 (Casq2) were increased in both muscles, while none of the other muscle contraction genes with changed expression were in common. For both muscles together muscle contraction gene expression was increased more than decreased (total 9 vs. 3 genes).

Of the 20 genes from the ion transport GO groups in the diaphragm with changed expression due to diabetes, 9 are involved in calcium transport (Table 3). Five calcium genes were increased (Myl6b, Casq2, Itga7, Cacnb2 and Sln) whereas four of the calcium genes were decreased (Pvalb, Pln, S100a3 and Gpd2). The other smaller groups of ion transport genes were sodium and/or potassium (3 genes), chloride (1 gene), water (1 gene) and zinc (1 gene) and several other miscellaneous groups. There were also 4 collagen genes listed in the ion GO groups. Of the 23 genes from the ion transport GO groups in the sternohyoid with diabetes-induced changed expression, 10 are involved in calcium transport (Table 3). Half of the calcium GO group genes increased (Casq2, S100a4, Mgl1, C1qb and C1qa) while the other half decreased (Fstl1, Eef2k, Atp2b3, Myl6b and Gpd2). The other smaller groups of ion transport genes are sodium and/or potassium (3 genes), magnesium (2 genes), and several other miscellaneous groups. There were also 3 collagen genes listed in the ion GO group. Among specific genes, three had altered expression in both muscles: Casq2 was increased and Gpd2 was decreased in both tissues while Myl6b was increased in diaphragm and decreased in sternohyoid. Not including the collagen genes, the ion transport genes were equally divided between increasing and decreasing expression with diabetes in both muscles (total 19 vs. 17 genes, not including collagen).

The collagen GO groups in the diaphragm had 3 genes and the sternohyoid had 4 genes with altered expression by diabetes. Col1a1 and Col1a2 had changed expression in both tissues. For both muscles all collagen gene expression changes were exclusively decreased (total 7 vs. 0).

Two sets of GO groups were over-represented in the diaphragm but not the sternohyoid muscle (Table 3). The blood circulation GO groups had 2 genes with increased and 3 genes with decreased expression. The oxidoreductase activity GO group had 4 genes with increased and 6 genes with decreased expression.

Two other sets of GO groups were over-represented in the sternohyoid but not the diaphragm muscle (Table 3). The immune and inflammatory GO groups had more genes with increased than decreased expression (total 7 vs 3). Of note is that all 5 complement genes had increased expression. The response to stress and wounding GO groups had 10 genes with increased expression and 7 genes with decreased genes expression. A subset of these genes were also included in the immune and inflammatory GO groups, including the 5 complement components with increased expression. However there were 10 genes in the stress and wounding GO groups that were not included in the immune and inflammatory GO groups.

**Figure 1:**
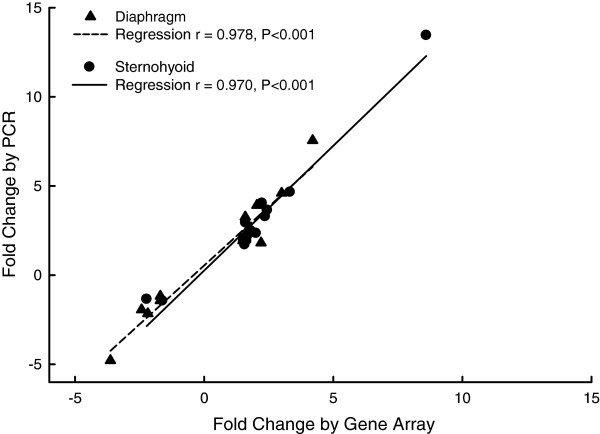
Relationship between fold changes in gene expression measured by gene expression microarray and real-time PCR.

To confirm changes in gene expression in diaphragm and sternohyoid, high throughput RT-PCR was performed on a subset of genes. The results which confirmed gene expression microarray data are presented Table 4. The direction of changes determined by PCR were in the same direction as that determined by expression arrays. There was a good and statistically significant correlation between the magnitude of altered expression measured by gene expression array and that measured by RT-PCR for these genes (Figure 1).

**Table 4:** Confirmatory results for changes in gene expression in diaphragm and sternhyoid measured by real-time PCR

***DIAPHRAGM***				
***Gene symbol***	***Fold change by microarray***	***Fold change by PCR***	***P value by PCR***	
Csrp3	4.2	7.6	0.022	
Mybph	3.0	4.6	0.0010	
Decr1	2.0	3.9	<0.001	
Sln	1.6	3.3	0.037	
Dbi	1.7	2.6	0.013	
Adfp	1.6	2.1	<0.001	
Kcnma1	1.5	2.1	<0.001	
Crot	1.6	2.0	<0.001	
Lrp1	1.5	1.9	0.004	
Prkaa1	2.2	1.8	<0.001	
Gpd2	−1.7	−1.2	0.010	
Acsl6	−1.7	−1.4	0.0060	
Pln	−2.4	−1.9	0.017	
Apln	−2.2	−2.2	<0.001	
Myh4	−3.6	−4.8	0.01	
***STERNOHYOID***				
***Gene symbol***	***Fold change by microarray***	***Fold change by PCR***	***P value by PCR***	
Decr1	3.3	4.7	<0.001	
Cdkn1a	2.4	3.7	0.0090	
Acot2	2.4	3.3	0.0060	
Fcgr2b	2.2	4.0	<0.001	
C4a	2.0	2.4	<0.001	
Adn	1.8	2.5	0.0060	
Cpt1b	1.7	2.2	0.0050	
Kcnma1	1.6	1.9	0.0030	
Crot	1.6	2.9	0.0020	
C1qa	1.6	2.0	0.038	
Cfh	1.6	1.7	0.0010	
Cpt2	1.5	2.1	0.0040	
Slc27a1	1.5	2.1	0.023	
Acsl6	−1.6	−1.4	0.0020	

Fold changes (FC) in gene expression are represented in the last column.

## Discussion

### Lipid and carbohydrate metabolism

The pattern of carbohydrate and lipid substrate use is regulated closely to meet the metabolic demands of muscles at rest and during exercise and furthermore plays important modulatory roles in the pathophysiology of disease states such as diabetes. There is extensive biochemical literature indicating that diabetes results in a shift in cellular energetics away from carbohydrate and towards lipid metabolism. Diabetic diaphragm has reduced uptake and phosphorylation of glucose, phosphorylation of fructose-6-phosphate, glycoysis, oxidation of pyruvate and acetate, uptake of acetoacete, production of glycogen, the proportion of the active complex of pyruvate dehydrogenase, and activites of hexokinase, phosphorylase and phosphofructokinase [20-27]. In addition, diabetic diaphragm has increased fat metabolism, uptake and oxidation of free fatty acids, output of glycerol, capacity for mobilization of intracellular lipids and intracellular concentrations of triglycerides, free fatty acid and long-chain fatty acyl-CoA [27-30]. In type I diabetic rat heart glucose uptake and oxidation decreases, while fatty acid metabolism increases, indicating that diabetes shifts the pattern of cardiac energy metabolism in the same direction as the diaphragm [29,31]. Gerber et al. [32] has previously found that long chain fatty acids are the major energy source in streptozotocin-induced type I diabetic cardiac muscle with their beta-oxidation in mitochondria generating nearly 70% of the ATP. The gene expression changes which contribute to the carbohydrate to lipid metabolic shift have only been partially elucidated. In streptozotocin-induced diabetic rat diaphragm (a type 1 diabetes model), we found a small increase in expression of genes involved in lipid metabolism and a large decrease in expression of genes involved in carbohydrate metabolism, indicating that the gene expression contribution to the carbohydrate to lipid metabolic shift is directed most strongly at changes in carbohydrate metabolism [12] In contrast, type 1 diabetic rat heart has no significant change in carbohydrate gene expression but substantially augmented gene expression related to lipid metabolism [14]. The findings of the present study indicate that in diaphragm and sternohyoid muscles type 2 diabetes produces a similar overall shift favoring carbohydrate over lipid metabolism gene expression that was seen in type 1 diabetic rat diaphragm. However, data from the current and previous studies indicate that there are considerable differences between type 1 and type 2 diabetes (Tables 5 and 6), as well as between diaphragm and sternohyoid, in the number of genes with changed expression, the magnitude of the expression changes, and in the identity of the specific genes involved.

**Table 5:** Specific Gene Ontology groups and number of genes which were examined in more detail in type II diabetic diaphragm and the corresponding changes in genes in type I diabetic diaphragm in a previous study (12)

***GO group category & specific GO term***	***Diaphragm***		***Diaphragm***	
	***Type II***		***Type I***	
	***# genes***	***P value***	***# genes***	***P value***
***Metbolism - Lipid***				
Lipid metabolic process	9	0.019		
Long-chain fatty acid transport	2	0.048		
Cellular lipid metabolic process				
Fatty acid metabolic process				
Lipid transport				
Fatty acid transport				
***Metabolism - Carbohydrate***				
Carbohydrate metabolic process	7	0.04	8	0.000061
Monosaccharide metabolic process	6	0.0037	5	0.0059
Hexose metabolic process	6	0.0036	5	0.0059
Cellular carbohydrate metabolic process	6	0.034	5	0.005
Carbohydrate biosynthetic process	4	0.016		
Glycerol-3-phosphate dehydrogenase complex				
Glycerol-3-phosphate dehydrogenase activity				
***Muscle contraction***				
Sarcomere	4	0.0066		
Myofibril	4	0.01		
Contractile fiber part	4	0.01		
Contractile fiber	4	0.014		
Striated muscle contraction	3	0.014		
Muscle system process				
Muscle contraction				
Regulation of muscle contraction				
***Ion transport***				
Ion transport	12	0.0015		
Calcium ion binding	9	0.024	10	0.00003
Inorganic anion transport	5	0.0072		
Anion transport	5	0.015		
Regulation of ion transport	3	0.014		
Channel activity	6	0.031		
Magnesium ion binding				
***Collagen***				
Fibrillar collagen	3	0.0015	3	0.00033
Collagen	3	0.018	4	0.00014
Collagen type I	2	0.013		
***Blood circulation***				
Blood circulation	5	0.016		
Circulatory system process	5	0.017		
***Oxidoreductase activity***				
Oxidoreductase activity	10	0.035	8	0.024

**Table 6:** Genes with changed expression in diaphragm of type II and type I (12) diabetic rats that were assigned to specific statistically over-represented gene ontology (GO) terms

**TYPE II DIAPHRAGM**		
	***Gene***	***Fold***
***Oxidoreductase activity***	***Symbol***	***Change***
**2,4-dienoyl CoA reductase 1, mitochondrial**	**Decr1**	**2**
IMP (inosine monophosphate) dehydrogenase 2	Impdh2	1.6
Potassium large conductance calcium-activated channel subfamily M, alpha member 1	Kcnma1	1.5
Solute carrier family 30 (zinc transporter), member 4	Slc30a4	1.5
Coenzyme Q7 homolog, ubiquinone (yeast)	Coq7	−1.6
Glycerol-3-phosphate dehydrogenase 2, mitochondrial	Gpd2	−1.7
S100 calcium binding protein A3	S100a3	−1.7
Dicarbonyl L-xylulose reductase	Dcxr	−2
Phytanoyl-CoA dioxygenase domain containing 1	Phyhd1	−2.1
Transmembrane 7 superfamily member 2	Tm7sf2	−2.5
***Carbohydrate metabolism***		
protein kinase, AMP-activated, alpha 1 catalytic subunit	Prkaa1	2.2
UDP-glucose pyrophosphorylase 2	Ugp2	−1.5
Solute carrier family 2 (facilitated glucose transporter), member 4	Slc2a4	−1.5
Coenzyme Q7 homolog, ubiquinone (yeast)	Coq7	−1.6
6-phosphofructo-2-kinase/fructose-2,6-biphosphatase 1	Pfkfb1	−1.7
**Glycerol-3-phosphate dehydrogenase 2, mitochondrial**	**Gpd2**	**−1.7**
Dicarbonyl L-xylulose reductase	Dcxr	−2
***Collagen***		
**Collagen, type I, alpha 2**	**Col1a2**	**−1.6**
**Collagen, type III, alpha 1**	**Col3a1**	**−1.7**
**Collagen, type I, alpha 1**	**Col1a1**	**−2**
Ficolin (collagen/fibrinogen domain containing) 1	Fcn1	−2.1
TYPE I DIAPHRAGM		
***Oxidoreductase activity***		
Cytochrome P450, family 2, subfamily e, polypeptide 1	Cyp2e1	6
Flavin containing monooxygenase 3	Fmo3	2.9
Crystallin, lamda 1	Cryl1	2.4
Lysyl oxidase	Lox	2.3
Ceruloplasmin	Cp	2.2
**2,4-dienoyl CoA reductase 1, mitochondrial**	**Decr1**	**2.2**
Aldehyde oxidase 1	Aox1	2.1
P450 (cytochrome) oxidoreductase	Por	2.1
***Carbohydrate metabolism***		
Neuraminidase 2	Neu2	−8.5
Phosphofructokinase, liver, B-type	Pfkl	−3.7
Solute carrier family 37 (glycerol-6-phosphate transporter), member 4	Slc37a4	−2.5
**Glycerol-3-phosphate dehydrogenase 2**	**Gpd2**	**−2.3**
Amylase 1, salivary	Amy1	−2.3
Phosphoglycerate mutase 2	Pgam2	−2.1
Lactate dehydrogenase A	Ldha	−2
Phosphoglucomutase 1	Pgm1	−2
Dihydrolipoamide S-acetyltransferase	Dlat	−2
***Collagen***		
**Collagen, type III, alpha 1**	**Col3a1**	**−3.7**
**Collagen, type 1, alpha 1**	**Col1a1**	**−3.5**
**Procollagen, type I, alpha 2**	**Col1a2**	**−3.2**
Fibrillin 1	Fbn1	−2.8
Secreted acidic cysteine rich glycoprotein	Sparc	−2.7
Collagen, type V, alpha 1	Col5a1	−2.4
Collagen, type V, alpha 3	Col5a3	−2.2

Fold changes in gene expression are represented in the last column. Genes in **bold** are common between type II and type I.

**Figure 2:**
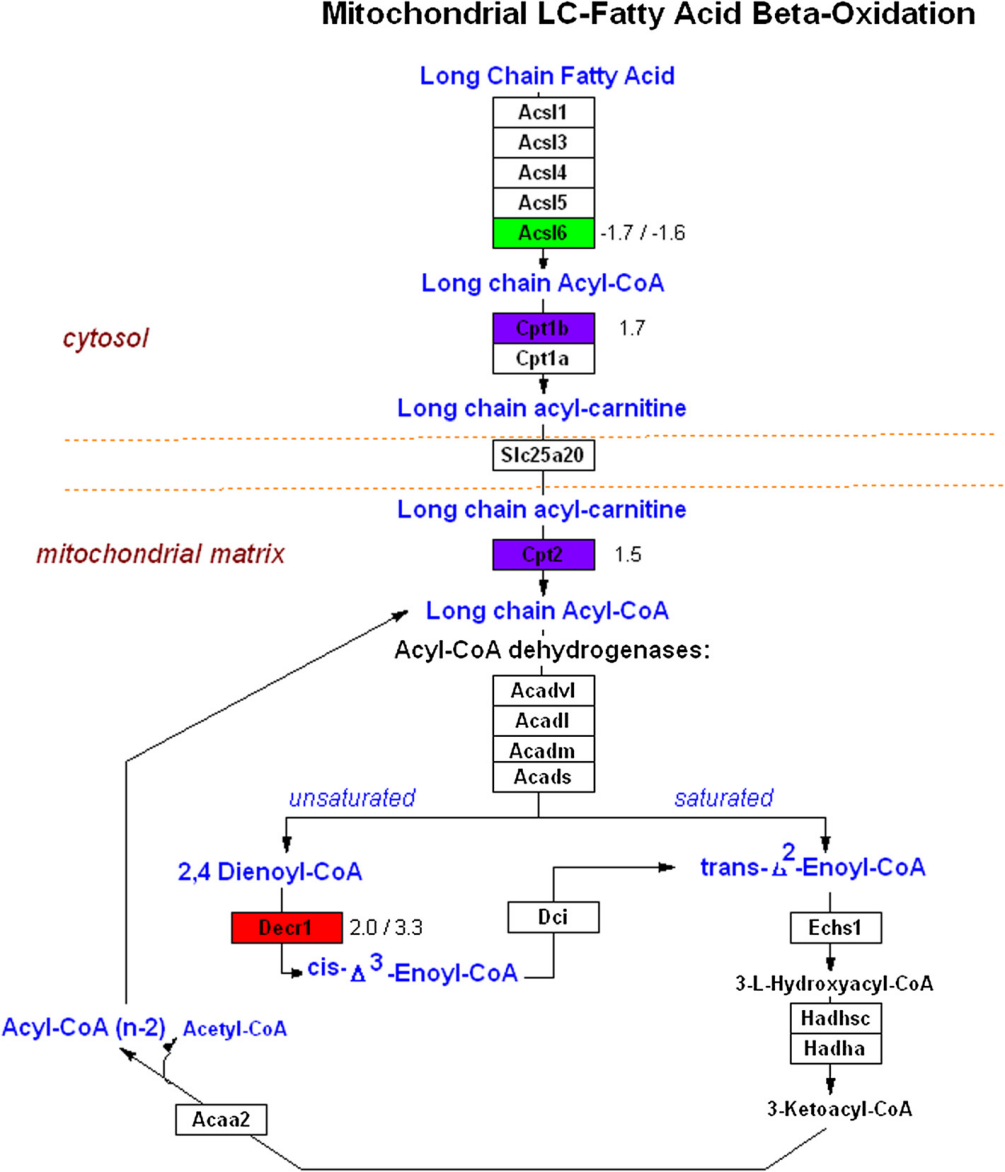
**Genes with changed expression in diabetic diaphragm and diabetic sternohyoid muscle that are involved in specific steps of fatty acid β-oxidation.** Genes with increased expression in both muscles diaphragm are indicated in red; genes with decreased expression in both muscles are indicated in green. Genes with increased expression in sternohyoid only are indicated in purple. Numbers indicate fold changes in diaphragm/sternohyoid.

In the present study there were two metabolism genes with decreased expression in both the diaphragm and sternohyoid. The first gene was acyl-CoA synthetase long-chain family member 6 (Acsl6) which catalyzes the ligation of long chain fatty acids with coenzyme A to produce long chain acyl-CoAs (Figure 2). This gene also had decreased expression in streptozotocin-induced diabetic diaphragm [12] and heart [33]. Acetyl-CoA is converted to malonyl-CoA which in turn inhibits CTP1 and the transport of fatty acid into the cell [34]. The second metabolism gene with decreased expression in both muscles was thyroid hormone responsive (Thrsp), which is believed to be involved in lipogenesis [35,36]. The diaphragm had decreased expression of transmembrane 7 superfamily member 2 (Tm7sf2) which is involved in cholesterol biosynthesis, [37] while the sternohyoid had a decrease in sterol regulatory element binding transcription factor 1 (Srebf1) which regulates the transcription of genes important for sterol biosynthesis. Srebf1 also had decreased expression in limb muscle of 12-week old type 2 diabetic rat [11].

There were several genes with increased expression in the lipid metabolism GO group that increased in previous studies of diabetes. 2,4-dienoyl CoA reductase 1 (Decr1) catalyzes the conversion of 2,4 dienoyl-CoA to cis-Δ^3^-enoyl-CoA and is involved in the mitochondrial long-chain fatty acid beta-oxidation pathway (Figure 2). In previous studies, Decr1 increased 5-fold in type 1 streptozotocin diabetic rat liver mitochondia [38], 2-fold in our previous studies in type 1 diabetic rat diaphragm [12] and heart [14], 2-fold in type 1 diabetic rat heart [39] and nearly four-fold in limb skeletal muscle of 12 week old type 2 diabetic rats [11]. Adipose differentiation related protein (Adfp) has increased expression in db/db mouse kidney [40]. Cell death-inducing DNA fragmentation factor (Cidea), also increased in the diabetic diaphragm, may play a role in lipolysis, but its role is still not clearly defined. In previous studies Cidea-null mutants have been diabetes-resistant [41,42]. It is possible that Cidea functions by modulating fatty acid metabolism since the Cidea-null mutants had much lower concentrations of plasma FFA and triglycerides [42].

In the sternohyoid, four out of the six lipid metabolism genes with increased expression (Cpt1b, Cpt2, Acot2 and Slc27a1) are also involved directly in fatty acid transport and oxidation. Carnitine palmitoyltransferase (Cpt1b) catalyses the transfer of long chain fatty acids to carnitine for translocation across the mitochondrial inner membrane and then Cpt2 is an inner mitochondrial membrane protein that converts long chain acylcarnitine to long chain acyl-CoA (Figure 2). They are also increased in streptozotocin-induced diabetic rat heart [32]. Cpt1b has heterogeneous changes, depending on tissue type. Cpt1b expression is increased in human type 2 diabetic adipose tissue [43] and type 1 diabetic rat heart [39]. However, it is reduced in human type II vastus lateralis [43] and streptozotocin-induced diabetic rat liver [44]. Acyl-CoA thioesterase 2 (Acot 2 or Mte1), catalyzes the hydrolysis of fatty acyl-CoA molecules into nonesterified fatty acid anions and free CoA in the mitochondria and has increased expression in heart and soleus of streptozotocin-induced diabetic rats [45]. Slc27a1 is a fatty acid transporter, which increases fatty acid supply when its expression is increased, and therefore is thought to increase fatty acid metabolism [46].

There were only two genes that had significantly decreased expression levels in the sternohyoid carbohydrate metabolism GO group (Gpd1 and Gpd2). Gpd2 expression was also decreased in diaphragm muscle. Gpd1 and Gpd2 are glycerol-3-phosphate dehydrogenase genes that are important members of the glycerol phosphate shuttle which are involved in the interconversion of glycerol-3-phosphate and dihydroxyacetone phosphate with concomitant reduction of FAD. Gpd2 also had decreased expression in the streptozotocin-induced diabetic rat heart [14] and diaphragm [12].

In addition to Gpd2, there were five other genes with decreased expression in the diaphragm that are involved in carbohydrate metabolism (Slc2a4, Ugp2, Dcxr, Pfkfb1, Coq7). Slc2a4, Glucose Transporter 4, is involved in transporting glucose across the membrane [47] and has diminished expression and function in type II diabetic rat heart [48] and slow muscle fibers and omental fat of type II diabetic patients [49,50]. Ugp2, UDP-glucose pyrophosphorylase 2 is essential for sucrose and polysaccharide synthesis [51] and has decreased expression in limb muscle of 12-week old type 2 diabetic rats [11]. The remaining 3 decreased diaphragm carbohydrate metabolism genes, Dcxr, Pfkfb1 and Coq7, were not significantly changed in any previous diabetes studies. Dicarbonyl L-xylulose reductase (Dcxr) functions in the metabolism of glucose [52]. 6-phosphofructo-2-kinase (Pfkfb1) is a rate limiting enzyme of glycolysis [53-57] which catalyzes the synthesis and degradation of fructose 2,6-bisphosphate. Coq7, coenzyme Q7, is a component of the electron transport chain which generates energy in the form of ATP.

### Muscle contraction

There has been a paucity of muscle contraction genes found to be altered due to diabetes in previous gene array studies. We are not aware of any muscle genes that were changed in the sternohyoid that have been found to be changed previously. However, the expression of cysteine and glycine-rich protein (Csrp3) gene increased in calf muscle in streptozotocin-induced diabetic mice, [58] similar to the diaphragm present study. This gene is thought to play a role in myogenesis. Mybph and Casq2 were the 2 genes that were increased in both muscles in the present study. Mybph is a skeletal muscle binding protein which binds myosin and is probably involved in the interaction with thick myofilaments in the A-band. Casq2 is a calcium binding protein that stores calcium for muscle contraction.

### Ion channels and transport

In our previous two studies of streptozotocin-induced type I diabetic heart and diaphragm gene expression we found decreased expression in 13 calcium binding genes in heart [14] and 10 calcium ion genes in the diaphragm [12].

Similar to the diabetic diaphragm in the present study, there was decreased expression of parvalbumin (Pvalb) in the nerve, [59] gastrocnemius [8] and diaphragm [12] of streptozotocin-induced type I diabetic rats. This protein binds two calcium ions and is involved in muscle relaxation. Previous studies have found conflicting results in levels of phospholamben (Pln) expression in diabetes. Pln is a key regulator of the sarcoplasmic reticulum ATPase and thus involved in calcium handling [60]. An increase in the mRNA and phospholamben protein levels is postulated to cause an increase in sarcoplasmic reticulum calcium reuptake inhibition [61]. In contrast to the current study, Zhong et al. [62] found a 31-60% increase in Pln in 4 and 6-week old streptozotocin-induced type I diabetic rat heart [62]. There was one previous study that found decreased Pln expression in streptozotocin-induced type I diabetic rat heart [63]. They also measured the amount of Pln phosphorylation by CaMK and PKA and found that to also be decreased and therefore postulated that because Pln phosphorylation inhibits the Pln inhibitory action, the decreased amount of Pln was still able to cause impaired Ca uptake. Glycerol-3-phosphate dehydrogenase (Gpd2) enhances lipid metabolism by binding calcium. Expression of this protein was decreased in streptozotocin-induced diabetic rat heart and diaphragm, [12,14] similar to the current study. The last decreased calcium binding gene in this group, S100 calcium binding protein A3 (S100a3), has not changed in any previous experiments with diabetes. The five increased calcium genes (Myl6b, Casq2, Itga7, Cacnb2 and Sln) have not had changed expression in previous diabetes studies. Their functions involve calcium binding and calcium channels. Sarcolipin (Sln) is also involved in sarcoplasmic reticulum calcium regulation similar to phospholamben, so it is possible that the increase in Sln expression could be a compensatory mechanism for the decrease in Pln.

In the diabetic sternohyoid, three of the five calcium channel genes with decreased expression (Fstl1, Atp2b3, Eef2k, Gpd2, Myl6b) were decreased in previous diabetes studies. Follistatin-like 1 (Fstl1) and glycerol-3-phosphate dehydrogenase (Gpd2) were decreased in streptozotocin-induced type 1 diabetic heart [14]. Atp2b3 protein content was decreased in insulin-resistant Wistar rat islet plasma membranes [64]. Several eukaryotic translation initiation and elongation factors are decreased in streptozotocin-induced diabetic rodent gastrocnemius muscle [8,65], however until the present study eukaryotic elongation factor kinase (Eef2k) has not previously been significantly changed due to diabetes. Eef2k is completely dependent on calcium and calmodulin and provides a key link between cellular energy status and the inhibition of protein synthesis [66-68]. Myl6b has not been significantly changed in previous studies involving diabetes. Five calcium genes were increased in the sternohyoid (Casq2, S100a4, Mgl1, C1qb and C1qa). The S100a4 gene was upregulated in a previous study in the peripheral leukocytes of streptozotocin-induced diabetic rats [69], while the other 4 did not have changed expression levels due to diabetes.

There are several genes that were classified in the ion channel GO grouping in the diaphragm that are involved in other channels besides calcium ions. These genes are Fxyd7, Scn3b and Kcnma1 which are involved in sodium and potassium channels, Clcn4-2, which is a voltage-gated chloride channel protein, Aqp1, which is a water channel, and Slc30a4, which is a zinc transporter. Lrp1 is a transmembrane receptor which functions in the endocytosis of over 40 structurally and functionally distinct ligands [70,71]. Aquaporin 1 (Aqp1) is the only non-calcium ion gene that has previously been examined in diabetes, however with conflicting results. Baelde et al. [72] found an increase in Aqp1 in human type II diabetic kidney, while others found no changes in protein levels in kidneys of streptozotocin-induced diabetic mice [73] and streptozotocin-induced diabetic rats [74].

There are also several genes that were classified in the ion channel GO grouping in the sternohyoid that are involved in other ion channels besides calcium. These genes are Fxyd2, Atp1b2 and Kcnma1 which are involved in sodium and potassium channels, Impa2 and Acsl6, which are regulated by magnesium, Slc16a3, which transports monocarboxylate, Amy1a, which binds bicarbonate, Chrnd, which binds acetylcholine, and Ltbp1, which binds TGF-B in order to regulate several collagens. Similar to the present study, Fxyd2, a Na/K ATPase regulator, has increased in human type II diabetic kidneys [72]. Changes in Atp1b2 and Kcnma1 expression have not been found in previous studies. In the present study, Impa2 increased and Acsl6 decreased. However in previous studies, the expression of Impa2 was decreased in type 2 genetically-affected (OLETF) diabetic cornea [75] and the expression of Acsl6 was decreased in streptozotocin-induced diabetic rat diaphragm [12] and heart [33]. The remaining non-calcium ion channel genes in the sternohyoid, Slc16a3, Amy1a, Chrnd and Ltbp1 did not have changed expression in previous studies of diabetes.

### Collagen

In the present study there were several collagen genes which were decreased in type 2 diabetic diaphragm (Col1a1, Col1a2, Col3a1) and sternohyoid (Col1a1, Col1a2, Col5a1), similar to previous studies. In previous studies, all of these collagen genes were significantly decreased in streptozotocin-induced diabetic rat diaphragm with fold changes greater than 2.0 [12]. Col1a2, Col3a1 and Col5a1expression were also decreased in streptozotocin-induced diabetic rat heart [14]. Col1a1 had decreased expression in db/db type 2 diabetic mice [76] and streptozotocin-induced diabetic rat gastrocnemius [8]. Col1a2 was decreased in newly forming bone of streptozotocin-induced diabetic mice [77].

### Blood circulation

In the blood circulation group that had significant changes in only the diaphragm, there was only one gene that was not also listed in another group and therefore not mentioned yet. Apelin (Apln) plays a role in normal glucolipidic metabolism [78] and has had conflicting results in previous experiments. The results of the present study agree with the results previous results of low plasma levels in type II diabetic Chinese humans [79,80]. There have also been other reports of both increased and decreased Apln levels in patients with type 2 diabetes [79,81].

### Oxidoreductase

In a previous study in streptozotocin-induced diabetic rat diaphragm, we found an increased expression in genes related to oxidative stress [12]. In the present study, there were 3 genes that were not also listed in another GO group (Decr1, Impdh2, Phyhd1). Decr1 catalyzes the conversion of trans-2,3-didehydroacyl-CoA and NADP(+) into trans,trans-2,3,4,5-tetradehydroacyl-CoA and NADPH. Similar to the present study, we have previously found an increase in expression in streptozotocin-induced diabetic rat heart [14]. Others have also found a 5-fold stimulation of activity in the liver mitochondria of streptozotocin-induced diabetic rats [38] and an increased expression in limb skeletal muscle of Zucker diabetic fatty rats [11]. Impdh2 and Phyhd1 have not had changed expression in previous diabetic studies.

### Immune & inflammatory, and response to stress & wounding

**Figure 3:**
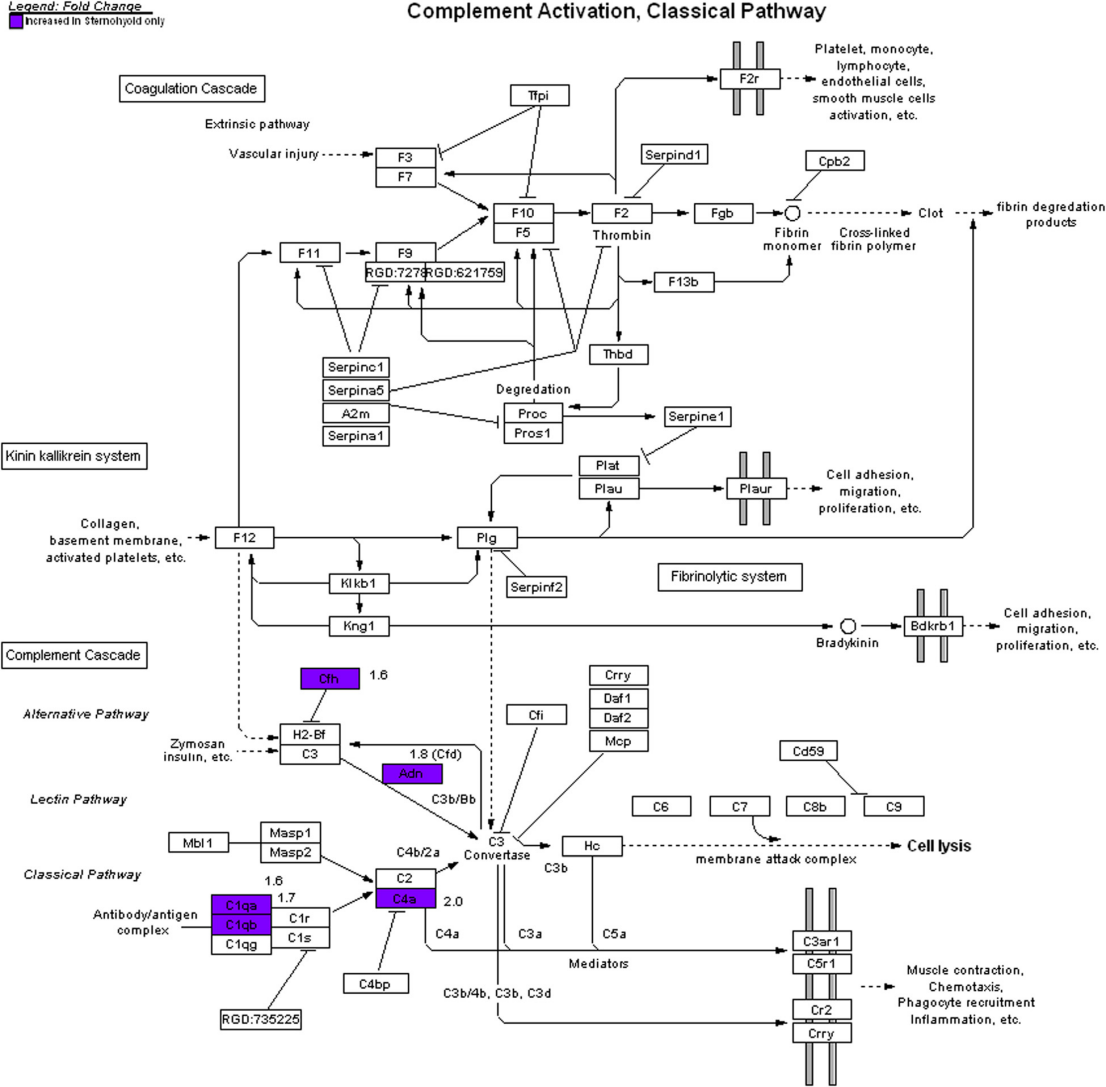
**Genes with changed expression in diabetic sternohyoid muscle that are involved in specific steps of the complement activation pathway.** Genes with increased expression in sternohyoid only are indicated in purple, with numbers indicating fold changes. There were no changes in complement-related gene expression changes in the diaphragm.

Most of the genes in these categories had increased expression in the diabetic sternohyoid, similar to a previous study in streptozotocin-induced diabetic liver [82]. However, these increases were completely absent in the diabetic diaphragm in the present study as well as type 1 diabetic diaphragm in our previous study^12^. Some of the sternohyoid genes with increased expression are depicted on the complement activation, classical pathway (Figure 3). One of the genes involved in the pathway is adipsin (Adn). Adipsin assembles with complement factor B to enzymatically cleave complement factor C3 to C3a-des-arg/ASP (acylation stimulating protein), which stimulates triglyceride production in adipose tissue [83]. It is the one complement component that has had changed expression due to diabetes in previous studies. Adipsin has increased in streptozotocin-induced diabetic mouse endothelium [83] and in streptozotocin-induced diabetic rat adipose tissue [84]. The complement factors (C1qb, C1qa, Cfh) are also involved in the complement activation classical pathway (Figure 3), but have not changed expression levels due to diabetes in previous studies. Cdkn1a is a cyclin-dependent kinase inhibitor [82]. It regulates cell division by arresting the cell cycle and is induced by oxidative stress [85,86]. Cdkn1a has increased expression in cardiac and soleus muscle of streptozotocin-induced diabetic rats [45] and liver of streptozotocin-induced diabetic mice [82].

All of the immune and inflammatory genes in the sternohyoid with increased expression are contained in the stress and wounding group too. The three genes with decreased expression (Dpp4, Mx1 and Spon2) are not contained in the stress and wounding group or any other of the significant GO groups in this study. Dipeptidyl peptidase IV (Dpp4) is a serine protease that exists on the surface of various types of cells and in a soluble form in plasma [87]. Circulating Dpp4 levels have been reported to be both increased [88,89] and decreased [90,91] in type 2 diabetic patients and increased in type I diabetic patients [88]. Dpp4 increases have been measured in many tissues of streptozotocin-induced diabetic rats [92]. Mx1 and Spon2 have had altered gene expression due to diabetes in any previous study.

## Conclusions

In conclusion, the current study shows that type 2 diabetes produces significant changes in gene expression of the diaphragm and sternohyoid muscles, many of which were not expected based on previous data on type 1 streptozotocin-induced diabetic diaphragm [12] as well as on both types 1 and type 2 diabetes in other muscle types [8-11,14,32,93]. The diaphragm had more gene expression decreases in carbohydrate metabolism due to diabetes than the sternohyoid, while the increases in lipid metabolism genes were similar in both muscles. Thus there was a larger metabolic gene expression shift in the diaphragm than the sternohyoid. However, for several other processes there were more closely shared magnitudes of gene expression changes (muscle contraction, ion transport, collagen). In addition, there were several gene expression changes in the diabetic sternohyoid that were not present in the diaphragm (immune & inflammatory, response to stress & wounding) and vice versa (oxidoreductase activity, blood circulation). The upper airway muscles and diaphragm therefore have targets in common as well as individual targets for future treatment strategies aimed at improving muscle function in diabetes and obstructive sleep apnea.

## Competing interests

The authors declare that they have no competing interests.

## Authors’ contributions

MM carried out studies, performed the statistical analysis and helped to draft the manuscript. EvL conceived of the study and participated in its design and coordination and helped to draft the manuscript. Both authors read and approved the final manuscript.

## Pre-publication history

The pre-publication history for this paper can be accessed here:

http://www.biomedcentral.com/1472-6823/13/43/prepub

## Supplementary Material

Additional file 1 **Complete list of genes with at least ±1.5-fold changed expression in diaphragm and sternohyoid of diabetic animals.** Fold changes (FC) in gene expression are represented in the last column. Click here for file
