# Supplementary material for: Gene expression of sternohyoid and diaphragm muscles in type 2 diabetic rats
Source: BMC Endocr Disord. 2013 Oct 7;13:43. doi: 10.1186/1472-6823-13-43 (PMC3851765; doi:10.1186/1472-6823-13-43)
Supplement: Additional file 1 — Complete list of genes with at least ±1.5-fold changed expression in diaphragm and sternohyoid of diabetic animals. Fold changes (FC) in gene expression are represented in the last column. [file 1472-6823-13-43-S1.doc]

**Additional file 1 - Complete list of genes with at least ±1.5-fold changed expression in diaphragm and sternohyoid of diabetic animals.**

Fold changes (FC) in gene expression are represented in the last column.

| **DIAPHRAGM** |  |  |
| --- | --- | --- |
| Gene Title | Gene Symbol | FC |
| myosin, light chain 6B, alkali, smooth muscle and non-muscle | Myl6b | 6.8 |
| cysteine and glycine-rich protein 3 | Csrp3 | 4.2 |
| phosphoinositide-3-kinase, class 2, gamma polypeptide | Pik3c2g | 4.1 |
| brain expressed gene 1 | Bex1 | 3.5 |
| serine (or cysteine) peptidase inhibitor, clade A, member 3N | Serpina3n | 3.2 |
| musculoskeletal, embryonic nuclear protein 1 | Mustn1 | 3.0 |
| myosin binding protein H | Mybph | 3.0 |
| enabled homolog (Drosophila) | Enah | 2.2 |
| protein kinase, AMP-activated, alpha 1 catalytic subunit | Prkaa1 | 2.2 |
| cell death-inducing DNA fragmentation factor, alpha subunit-like effector A | Cidea | 2.2 |
| sodium channel, voltage-gated, type III, beta | Scn3b | 2.2 |
| tumor necrosis factor receptor superfamily, member 12a | Tnfrsf12a | 2.1 |
| V-set domain containing T cell activation inhibitor 1 | Vtcn1 | 2.1 |
| regulator of G-protein signaling 7 | Rgs7 | 2.1 |
| 2,4-dienoyl CoA reductase 1, mitochondrial | Decr1 | 2.0 |
| receptor accessory protein 6 | Reep6 | 2.0 |
| adenosine monophosphate deaminase 3 | Ampd3 | 2.0 |
| N-myc downstream regulated gene 4 | Ndrg4 | 1.9 |
| glutathione S-transferase, mu 5 | Gstm5 | 1.9 |
| tumor protein D52-like 1 | Tpd52l1 | 1.9 |
| insulin-like growth factor 2 receptor | Igf2r | 1.9 |
| androgen-induced 1 | Aig1 | 1.8 |
| sulfide quinone reductase-like (yeast) | Sqrdl | 1.8 |
| Homeodomain interacting protein kinase 2 | Hipk2 | 1.8 |
| period homolog 2 (Drosophila) | Per2 | 1.8 |
| diazepam binding inhibitor (GABA receptor modulator, acyl-Coenzyme A binding protein) | Dbi | 1.7 |
| dopa decarboxylase (aromatic L-amino acid decarboxylase) | Ddc | 1.7 |
| suprabasin | Sbsn | 1.7 |
| calcium channel, voltage-dependent, beta 2 subunit | Cacnb2 | 1.6 |
| solute carrier family 35, member F5 | Slc35f5 | 1.6 |
| response gene to complement 32 | Rgc32 | 1.6 |
| proteolipid protein 2 (colonic epithelium-enriched) | Plp2 | 1.6 |
| flotillin 1 | Flot1 | 1.6 |
| sarcolipin | Sln | 1.6 |
| carnitine O-octanoyltransferase | Crot | 1.6 |
| PDZ and LIM domain 3 | Pdlim3 | 1.6 |
| lipopolysaccharide binding protein | Lbp | 1.6 |
| microtubule-associated protein 1A | Map1a | 1.6 |
| adipose differentiation related protein | Adfp | 1.6 |
| IMP (inosine monophosphate) dehydrogenase 2 | Impdh2 | 1.6 |
| torsin A interacting protein 2 | Tor1aip2 | 1.5 |
| ubiquitin specific peptidase 28 | Usp28 | 1.5 |
| calsequestrin 2 (cardiac muscle) | Casq2 | 1.5 |
| epoxide hydrolase 1, microsomal | Ephx1 | 1.5 |
| integrin alpha 7 | Itga7 | 1.5 |
| G protein-coupled receptor 155 | Gpr155 | 1.5 |
| G protein-coupled receptor associated sorting protein 1 | Gprasp1 | 1.5 |
| potassium large conductance calcium-activated channel, subfamily M, alpha member 1 | Kcnma1 | 1.5 |
| phosphatidic acid phosphatase type 2 domain containing 2 | Ppapdc2 | 1.5 |
| phosphodiesterase 3B, cGMP-inhibited | Pde3b | 1.5 |
| low density lipoprotein-related protein 1 (alpha-2-macroglobulin receptor) | Lrp1 | 1.5 |
| similar to RIKEN cDNA C430004E15 | LOC499749 | 1.5 |
| PHD finger protein 7 | Phf7 | 1.5 |
| solute carrier family 30 (zinc transporter), member 4 | Slc30a4 | 1.5 |
| HIG1 hypoxia inducible domain family, member 1A | Higd1a | -1.5 |
| solute carrier family 2 (facilitated glucose transporter), member 4 | Slc2a4 | -1.5 |
| tumor protein p53 inducible nuclear protein 2 | Trp53inp2 | -1.5 |
| UDP-glucose pyrophosphorylase 2 | Ugp2 | -1.5 |
| cytochrome b reductase 1 | Cybrd1 | -1.5 |
| aquaporin 1 | Aqp1 | -1.5 |
| histocompatibility 2, class II antigen E alpha | H2-Ea | -1.6 |
| 3-oxoacid CoA transferase 1 | Oxct1 | -1.6 |
| ectonucleotide pyrophosphatase/phosphodiesterase 2 | Enpp2 | -1.6 |
| DNA-damage-inducible transcript 4 | Ddit4 | -1.6 |
| collagen, type I, alpha 2 | Col1a2 | -1.6 |
| thiopurine S-methyltransferase | Tpmt | -1.6 |
| complement component 7 /// tubulin, beta 2c | C7 /// Tubb2c | -1.6 |
| coenzyme Q7 homolog, ubiquinone (yeast) | Coq7 | -1.6 |
| receptor accessory protein 1 | Reep1 | -1.7 |
| glycerol-3-phosphate dehydrogenase 2, mitochondrial | Gpd2 | -1.7 |
| dual-specificity tyrosine-(Y)-phosphorylation regulated kinase 2 | Dyrk2 | -1.7 |
| 6-phosphofructo-2-kinase/fructose-2,6-biphosphatase 1 | Pfkfb1 | -1.7 |
| poly(A) binding protein interacting protein 1 | Paip1 | -1.7 |
| FXYD domain-containing ion transport regulator 7 | Fxyd7 | -1.7 |
| S100 calcium binding protein A3 | S100a3 | -1.7 |
| acyl-CoA synthetase long-chain family member 6 | Acsl6 | -1.7 |
| REV3-like, catalytic subunit of DNA polymerase zeta (yeast) | Rev3l | -1.7 |
| collagen, type III, alpha 1 | Col3a1 | -1.7 |
| mitochondrial protein 18 kDa | Mtp18 | -1.7 |
| aspartoacylase | Aspa | -1.8 |
| bone marrow stromal cell antigen 2 | Bst2 | -1.8 |
| RT1 class II, locus Ba | RT1-Ba | -1.8 |
| chloride channel 4-2 | Clcn4-2 | -1.8 |
| 1-acylglycerol-3-phosphate O-acyltransferase 3 | Agpat3 | -1.9 |
| collagen, type I, alpha 1 | Col1a1 | -2.0 |
| sialidase 2 (cytosolic sialidase) | Neu2 | -2.0 |
| dicarbonyl L-xylulose reductase | Dcxr | -2.0 |
| parvalbumin | Pvalb | -2.0 |
| phytanoyl-CoA dioxygenase domain containing 1 | Phyhd1 | -2.1 |
| ficolin (collagen/fibrinogen domain containing) 1 | Fcn1 | -2.1 |
| thyroid hormone responsive | Thrsp | -2.1 |
| apelin | Apln | -2.2 |
| phospholamban | Pln | -2.4 |
| transmembrane 7 superfamily member 2 | Tm7sf2 | -2.5 |
| serine peptidase inhibitor, Kazal type 8 | Spink8 | -2.9 |
| ataxia, cerebellar, Cayman type | Atcay | -2.9 |
| myosin, heavy chain 4, skeletal muscle | Myh4 | -3.6 |
|  |  |  |
| **STERNOHYOID** |  |  |
| Gene Title | Gene Symbol | Mn FC |
| myosin binding protein H | Mybph | 8.7 |
| similar to C20orf95 | RGD1562428 | 3.9 |
| 2,4-dienoyl CoA reductase 1, mitochondrial | Decr1 | 3.3 |
| aquaporin 7 | Aqp7 | 2.7 |
| retinol binding protein 7, cellular | Rbp7 | 2.5 |
| carnitine O-octanoyltransferase | Crot | 2.5 |
| cyclin-dependent kinase inhibitor 1A (p21, Cip1) | Cdkn1a | 2.4 |
| tumor protein D52-like 1 | Tpd52l1 | 2.4 |
| acyl-CoA thioesterase 2 | Acot2 | 2.4 |
| nudix (nucleoside diphosphate linked moiety X)-type motif 7 | Nudt7 | 2.2 |
| Fc fragment of IgG, low affinity IIb, receptor (CD32) | Fcgr2b | 2.2 |
| lectin, galactoside-binding, soluble, 3 | Lgals3 | 2.1 |
| nudix (nucleoside diphosphate linked moiety X)-type motif 4 | Nudt4 | 2.1 |
| glutathione S-transferase, mu 5 | Gstm5 | 2.0 |
| calsequestrin 2 (cardiac muscle) | Casq2 | 2.0 |
| latent transforming growth factor beta binding protein 1 | Ltbp1 | 2.0 |
| complement component 4a | C4a | 2.0 |
| cholinergic receptor, nicotinic, delta | Chrnd | 1.9 |
| S100 calcium-binding protein A4 | S100a4 | 1.9 |
| deleted in malignant brain tumors 1 | Dmbt1 | 1.9 |
| retinol saturase (all trans retinol 13,14 reductase) | Retsat | 1.9 |
| glutathione S-transferase mu 2 | Gstm2 | 1.9 |
| similar to KIAA0564 protein | RGD1308772 | 1.8 |
| SAP domain containing ribonucleoprotein | Sarnp | 1.8 |
| Dickkopf homolog 2 (Xenopus laevis) | Dkk2 | 1.8 |
| adipsin | Adn | 1.8 |
| transmembrane protein 97 | Tmem97 | 1.8 |
| inositol (myo)-1(or 4)-monophosphatase 2 | Impa2 | 1.8 |
| microsomal glutathione S-transferase 1 | Mgst1 | 1.7 |
| membrane-spanning 4-domains, subfamily A, member 6B | Ms4a6b | 1.7 |
| complement component 1, q subcomponent, beta polypeptide | C1qb | 1.7 |
| lysozyme 2 | Lyz2 | 1.7 |
| GIPC PDZ domain containing family, member 2 | Gipc2 | 1.7 |
| carnitine palmitoyltransferase 1b, muscle | Cpt1b | 1.7 |
| abhydrolase domain containing 1 | Abhd1 | 1.6 |
| potassium large conductance calcium-activated channel, subfamily M, alpha member 1 | Kcnma1 | 1.6 |
| family with sequence similarity 69, member B | Fam69b | 1.6 |
| PDX1 C-terminal inhibiting factor 1 | Pcif1 | 1.6 |
| ribosomal protein L22 like 1 | Rpl22l1 | 1.6 |
| Nucleosome assembly protein 1-like 1 | Nap1l1 | 1.6 |
| complement component 1, q subcomponent, alpha polypeptide | C1qa | 1.6 |
| glutathione S-transferase A3 | Gsta3 | 1.6 |
| similar to potassium channel modulatory factor 1 | LOC684322 | 1.6 |
| DCP1 decapping enzyme homolog b (S. cerevisiae) | Dcp1b | 1.6 |
| complement factor H | Cfh | 1.6 |
| FXYD domain-containing ion transport regulator 2 | Fxyd2 | 1.6 |
| carnitine palmitoyltransferase 2 | Cpt2 | 1.5 |
| macrophage galactose N-acetyl-galactosamine specific lectin 1 | Mgl1 | 1.5 |
| placenta-specific 8 | Plac8 | 1.5 |
| solute carrier family 27 (fatty acid transporter), member 1 | Slc27a1 | 1.5 |
| low density lipoprotein receptor-related protein 6 | Lrp6 | -1.5 |
| Lix1 homolog (mouse)-like | Lix1l | -1.5 |
| follistatin-like 1 | Fstl1 | -1.5 |
| epidermal growth factor receptor | Egfr | -1.5 |
| SRY (sex determining region Y)-box 4 | Sox4 | -1.6 |
| torsin family 3, member A | Tor3a | -1.6 |
| cell division cycle associated 3 | Cdca3 | -1.6 |
| Similar to SH3-domain binding protein 3 | LOC688018 | -1.6 |
| 2,4-dienoyl CoA reductase 2, peroxisomal /// RAB11 family interacting protein 3 (class II) | Decr2 /// Rab11fip3 | -1.6 |
| serine (or cysteine) peptidase inhibitor, clade H, member 1 | Serpinh1 | -1.6 |
| glycerol-3-phosphate dehydrogenase 1 (soluble) | Gpd1 | -1.6 |
| heat shock protein, alpha-crystallin-related, B6 | Hspb6 | -1.6 |
| acyl-CoA synthetase long-chain family member 6 | Acsl6 | -1.6 |
| guanidinoacetate N-methyltransferase | Gamt | -1.6 |
| seven in absentia 2 | Siah2 | -1.6 |
| CXXC finger 5 | Cxxc5 | -1.6 |
| ATPase, Na+/K+ transporting, beta 2 polypeptide | Atp1b2 | -1.6 |
| solute carrier family 16, member 3 (monocarboxylic acid transporter 4) | Slc16a3 | -1.7 |
| similar to RCK | RGD1564560 | -1.7 |
| paired related homeobox 1 | Prrx1 | -1.7 |
| SET domain containing (lysine methyltransferase) 8 | Setd8 | -1.7 |
| amylase, alpha 1A (salivary) | Amy1a | -1.7 |
| phosphorylase kinase gamma 1 | Phkg1 | -1.8 |
| phosphoglycolate phosphatase | Pgp | -1.8 |
| GTPase, IMAP family member 4 | Gimap4 | -1.8 |
| prostaglandin F2 receptor negative regulator | Ptgfrn | -1.8 |
| glycerol-3-phosphate dehydrogenase 2, mitochondrial | Gpd2 | -1.8 |
| collagen, type V, alpha 1 | Col5a1 | -1.8 |
| regulator of chromosome condensation 2 | Rcc2 | -1.8 |
| collagen, type XV, alpha 1 | Col15a1 | -1.9 |
| A kinase (PRKA) anchor protein 12 | Akap12 | -1.9 |
| collagen, type I, alpha 1 | Col1a1 | -1.9 |
| dipeptidylpeptidase 4 | Dpp4 | -1.9 |
| myxovirus (influenza virus) resistance 1 | Mx1 | -1.9 |
| similar to chromosome 14 open reading frame 50 | RGD1309051 | -1.9 |
| leprecan-like 2 | Leprel2 | -1.9 |
| nuclear receptor subfamily 4, group A, member 3 | Nr4a3 | -1.9 |
| dual-specificity tyrosine-(Y)-phosphorylation regulated kinase 2 | Dyrk2 | -1.9 |
| spondin 2, extracellular matrix protein | Spon2 | -2.0 |
| similar to neuron navigator 1 | LOC685707 | -2.0 |
| eukaryotic elongation factor-2 kinase | Eef2k | -2.0 |
| heat shock protein 2 | Hspa2 | -2.0 |
| ATPase, Ca++ transporting, plasma membrane 3 | Atp2b3 | -2.1 |
| aspartoacylase | Aspa | -2.1 |
| thyroid hormone responsive | Thrsp | -2.1 |
| collagen, type I, alpha 2 | Col1a2 | -2.2 |
| microfibrillar-associated protein 4 | Mfap4 | -2.2 |
| TBC1 domain family, member 1 | Tbc1d1 | -2.3 |
| sterol regulatory element binding transcription factor 1 | Srebf1 | -2.4 |
| ataxia, cerebellar, Cayman type | Atcay | -2.5 |
| myosin, light chain 6B, alkali, smooth muscle and non-muscle | Myl6b | -2.5 |
| ribonucleotide reductase M2 | Rrm2 | -2.5 |
